# The fluorescence of a mercury probe based on osthol

**DOI:** 10.3762/bjoc.17.3

**Published:** 2021-01-05

**Authors:** Guangyan Luo, Zhishu Zeng, Lin Zhang, Zhu Tao, Qianjun Zhang

**Affiliations:** 1 Key Laboratory of Macrocyclic and Supramolecular Chemistry of Guizhou Province, Guizhou University, Guiyang 550025, China

**Keywords:** fluorescence probe, mercury, recognition mechanism

## Abstract

The ability of osthol (OST) to recognize mercury ions in aqueous solution was studied using fluorescence, UV–vis spectrophotometry, mass spectrometry, and ^1^H NMR spectroscopy, and the recognition mechanism is discussed. The results showed that OST and Hg^2+^ can form a complex with a stoichiometric ratio of 1:1. The binding constant was 1.552 × 10^5^ L∙mol^−1^, having a highly efficient and specific selectivity for Hg^2+^. The fluorescence intensity of OST showed a good linear correlation with the Hg^2+^ concentration (6.0 × 10^−5^ to 24.0 × 10^−5^ mol∙L^−1^, *R*^2^ = 0.9954), and the detection limit of the probe was 5.04 × 10^−8^ mol∙L^−1^, which can be used for the determination of Hg^2+^ traces.

## Introduction

Mercury is a dangerous heavy-metal pollutant. Inorganic mercury (Hg^2+^) can be transformed into methyl mercury (MeHg^+^) by sulfate-reducing bacteria [1–3]. MeHg^+^ can accumulate in organisms through the food chain, resulting in serious and irreversible nerve damage. Therefore, it is very important to develop a highly sensitive and selective method for mercury detection. At present, the detection of mercury mainly includes atomic absorption and atomic emission spectrometry [4], inductively coupled plasma mass spectrometry [5–6], and capillary electrophoresis [7]. However, the application of these analytical methods in mercury detection is limited due to the complex sample pretreatment procedures, expensive instruments, and other factors [8–9]. Fluorescent-probe instruments have been widely used due to the advantages of the simple operation, low cost, and high sensitivity [10–12]. In recent years, many fluorescent molecular probes have been reported. Because Hg^2+^ has a very strong quenching effect on fluorescence, most of the fluorescent molecular Hg^2+^ probes are of the fluorescence-quenching type and are easily interfered with by other quenching processes [13–17]. Fluorescence-enhanced probes have received wide attention because of their enhanced fluorescence signal, which can better exclude the influence of instrument noise and other factors, reduce measurement errors, and thus have a higher sensitivity. Osthol (OST) is a coumarin compound extracted from the fruit of *Cnidium monnieri* (L.) cuss [18–19]. Modern pharmacological studies have shown that OST has antihypertension, anti-epilepsy [20], anti-arrhythmia, anti-fatty-liver-disease, antitumor [21], antiosteoporosis, and other effects [22]. As a new type of fluorescence probe [23], OST as a probe for the detection of mercury has not been reported. In this paper, OST was used as a fluorescent probe since the fluorescence intensity of OST at 406 nm increased significantly when Hg^2+^ was added. The probe had a high selectivity and sensitivity for Hg^2+^ recognition and can be used for the quantitative detection and monitoring of mercury ions in the environment.

## Results and Discussion

### OST fluorescence probe for Hg^2+^ identification

#### Selectivity of the fluorescent OST probe to metal ions

**Figure 1:**
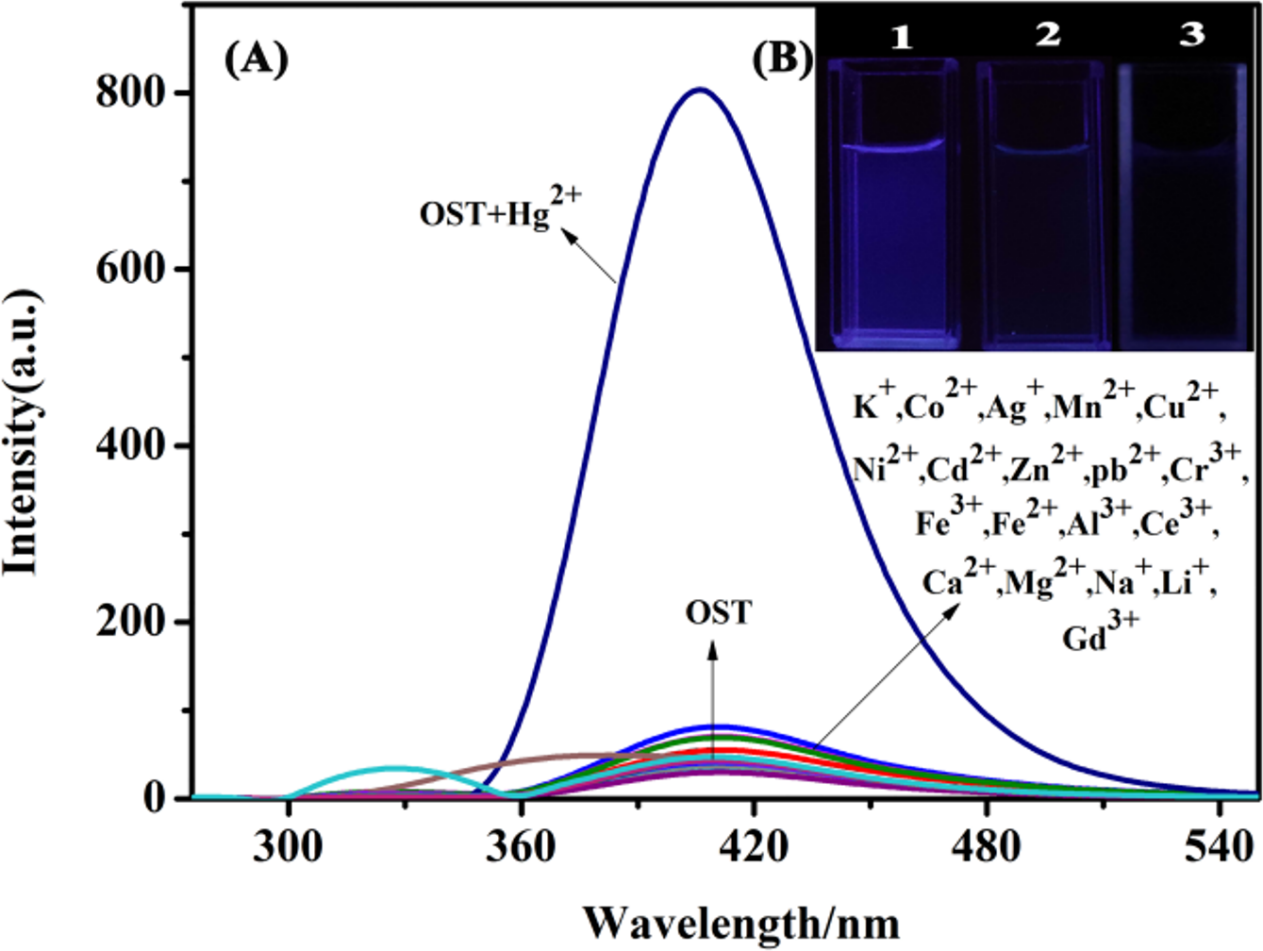
(A) Fluorescence spectra of OST (*c* = 3.0 × 10^−5^ mol∙L^−1^) upon the addition of various metal ions. (B) Fluorescent color change under ultraviolet light at 365 nm upon adding Hg^2+^ ions. 1: OST + Hg^2+^ solution, 2: OST + other ions, and 3: OST solution.

The specificity of a probe for metal ions is the key factor to evaluate the performance of fluorescent probes. As shown in Figure 1A, the selectivity of OST to common metal ions is monitored by the fluorescence spectrum (λ_ex_ = 325 nm). OST exhibits a weak fluorescence intensity at λ_ex_ = 406 nm. When adding different metal ions (Hg^2+^, Ca^2+^, Na^+^, Mg^2+^, Al^3+^, Cd^2+^, Cu^2+^, Pb^2+^, Ni^2+^, Co^2+^, Fe^3+^, Ag^+^, Cr^3+^, and other metal ions) in 10 times the equivalent concentration to the OST probe, only Hg^2+^ produced an obvious fluorescence enhancement (the intensity increased sharply by about 27 times), and the other metal ions had little effect on the fluorescence intensity of OST, which indicates that the probe had a high selectivity for Hg^2+^. At the same time, under irradiation with 365 nm ultraviolet light, it was found that the OST solution was colorless and transparent when metal ions other than mercury were added. Only after Hg^2+^ was added, it showed a bright blue fluorescence (Figure 1B). These obvious changes in the color and fluorescence intensity indicated that OST had a good Hg^2+^ recognition effect and achieved the visual detection of Hg^2+^.

#### Influence of coexisting ions on the detection of Hg^2+^

**Figure 2:**
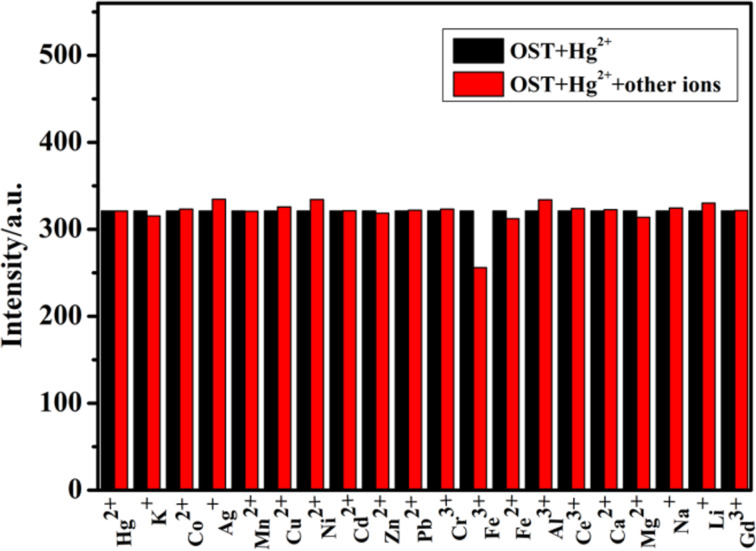
Metal ion selectivity of OST–Hg^2+^ (*c* = 3.0 × 10^−5^ mol∙L^−1^) in the presence of 1.2 × 10^−4^ mol∙L^−1^ of various metal ions (H_2_O/CH_3_OH 97:3, v/v, λ_ex_ = 325 nm).

To investigate the interference ability of coexisting ions with the determination of Hg^2+^, a coexisting ion experiment was carried out (Figure 2) [24]. The fluorescence intensity was measured by adding 1.2 × 10^−4^ mol∙L^−1^ of various metal ions to the OST–Hg^2+^ (*c* = 3.0 × 10^−5^ mol∙L^−1^) probe system. It is found that Fe^3+^ had a certain fluorescence quenching effect on the system due to the paramagnetism [25], but the fluorescence quenching effect was not enough to affect the recognition of mercury ions by the probe, and the presence of other coexisting metal ions did not affect the fluorescence spectrum change of the OST–Hg^2+^ system.

#### Drawing the standard curve [26]

**Figure 3:**
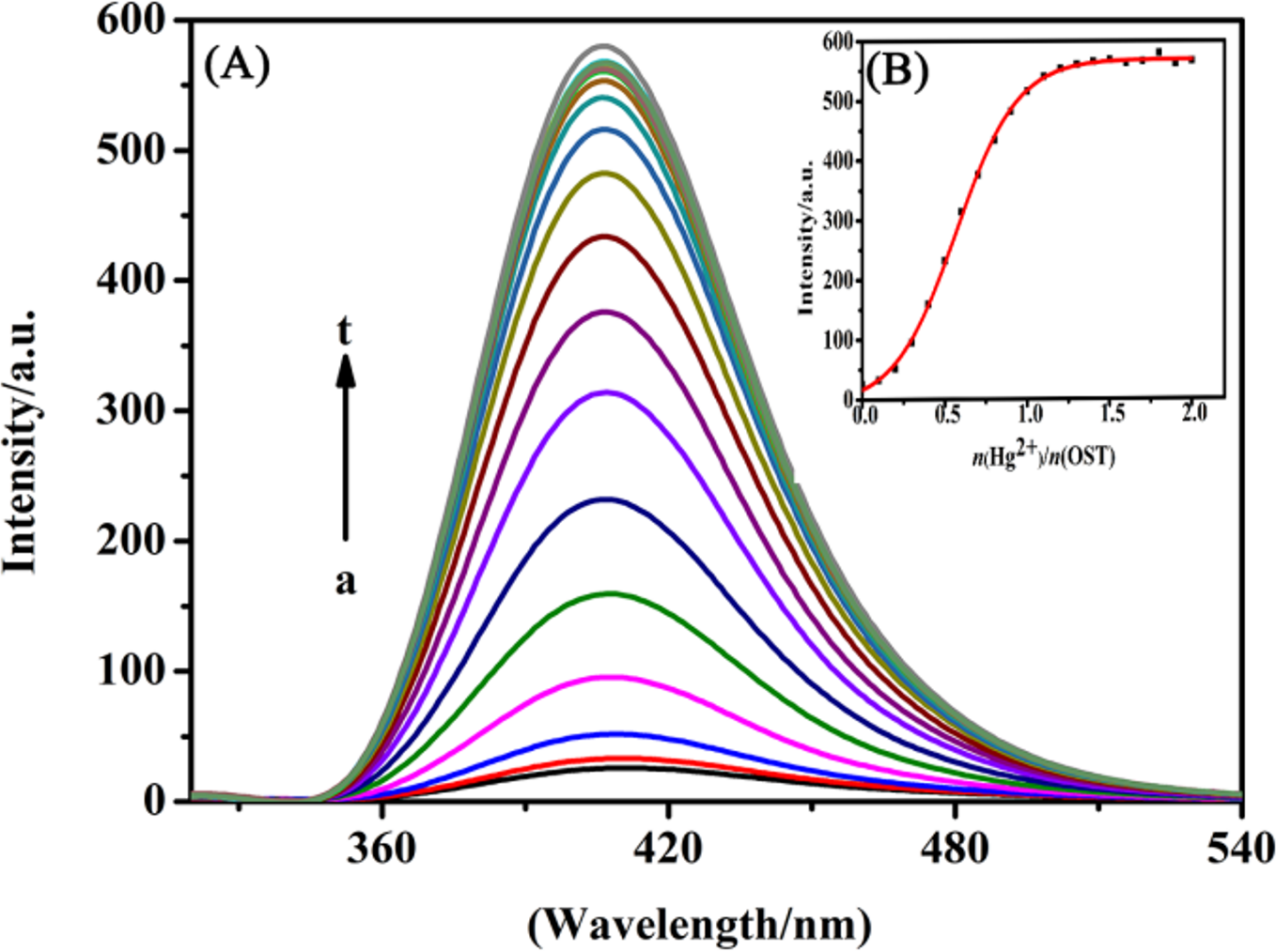
Fluorescence spectra of OST (*c* = 3 × 10^−5^ mol∙L^−1^) in H_2_O/CH_3_OH 97:3, v/v in the presence of an increasing concentration of Hg^2+^ ions (λ_ex_ = 325 nm). (A) *n*(Hg^2+^)/*n*(OST) = 0, 0.1, 0.2, 0.3, 0.4, 0.5, 0.6, 0.7, 0.8, 0.9, 1.0, 1.1, 1.2, 1.3, 1.4, 1.5, 1.6, 1.7, 1.8, 1.9, 2.0 (a→t). (B) Plot of the fluorescence intensity of OST at 406 nm.

**Figure 4:**
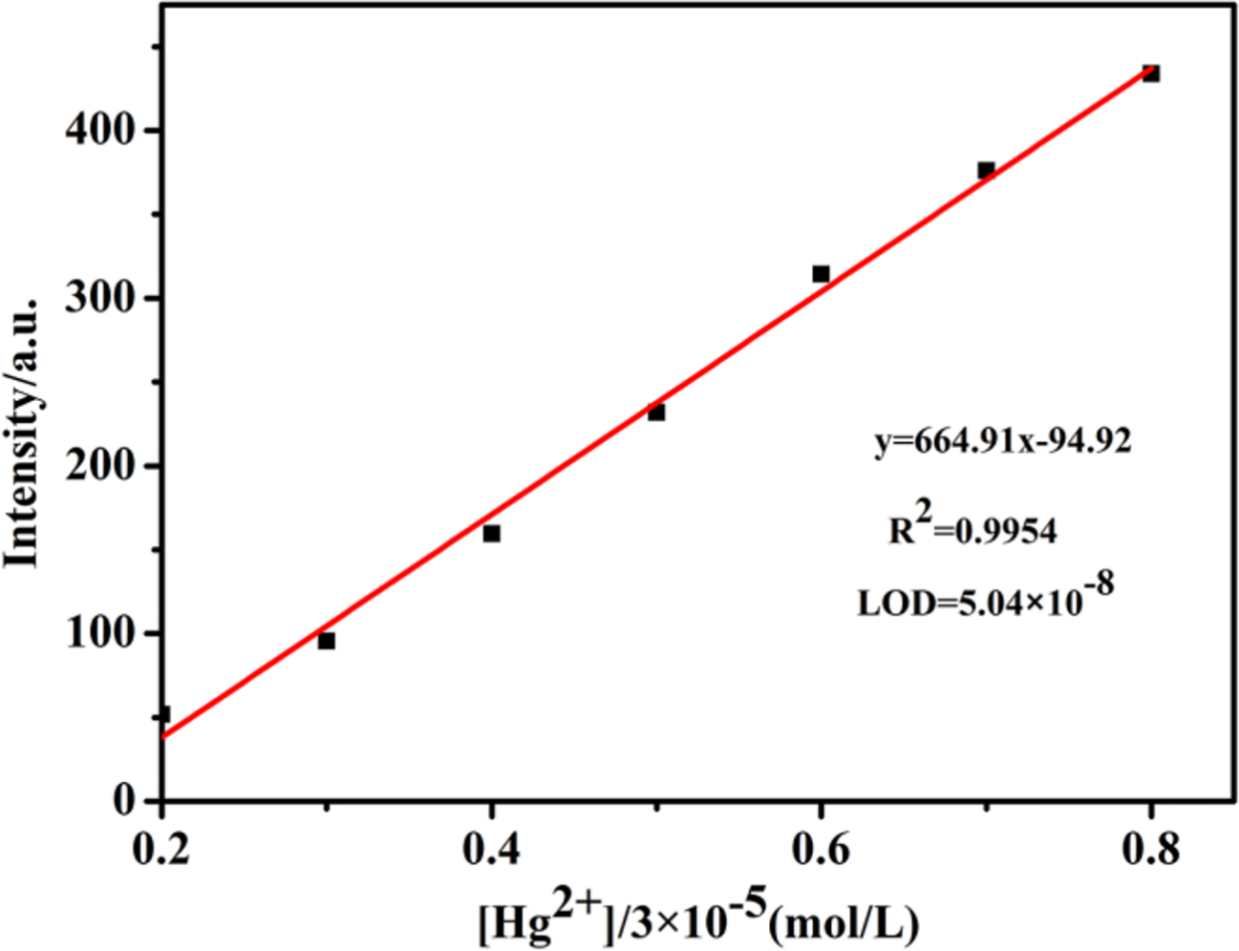
The standard Job curve of mercury ions to OST (*c* = 3.0 × 10^−5^ mol∙L^−1^) at 406 nm (pH 7.0, H_2_O/CH_3_OH 97:3, v/v, λ_ex_ = 325 nm).

Figure 3A shows the fluorescence spectrum (λ_ex_ = 325 nm) of the OST probe following interaction with Hg^2+^ at different concentrations. With the increase of the Hg^2+^ concentration, the fluorescence intensity at 406 nm showed an increase of the Hg^2+^ dependence. The fluorescence intensity reached the maximum when 1.0 equivalent of Hg^2+^ was added. When the molar Hg^2+^/OST ratio was 1:1, the fluorescence intensity increased by about 27 times, and a further increase of the Hg^2+^ concentration did not cause further changes in the fluorescence intensity (Figure 3B). The linear relationship between the magnitude of the increase in the fluorescence intensity and the concentration of the mercury ions was in the range of 6.0 × 10^−5^ to 24.0 × 10^−5^ mol∙L^−1^ (Figure 4). The linear regression equation was *y* = 664.91 ∙ *x* − 94.92, and the coefficient of determination was *R*^2^ = 0.9954. According to the formula for the limit of detection (LOD) = 3 ∙ σ/*K* (*n* = 7, where σ is the standard deviation and *K* is the slope of the calibration curve) [27], the detection limit of the OST probe for Hg^2+^ was 5.04 × 10^−8^ mol∙L^−1^.

#### Impact of the pH value of the solution

The effects of the pH value on the fluorescence intensity of the system are shown in Figure S1, Supporting Information File 1. The fluorescence intensity of the OST probe was weak in the pH value range of 2–10. When Hg^2+^ was added, the fluorescence intensity of OST–Hg^2+^ was strong, and stable in the pH value range of 4–10. Especially at pH 4–8.0, the fluorescence intensity reached the maximum value. Therefore, we chose pH 4–8.0 as the best measurement conditions.

#### Effect of the solvent

Figure S2 in Supporting Information File 1 shows the effects of different solvents on the fluorescence intensity of OST and OST–Hg^2+^. The results showed that the fluorescence intensity of OST was relatively weak in the studied solvents, but the fluorescence signal of OST was significantly enhanced after adding Hg^2+^, and the fluorescence intensity in different solvents was not very different. Because Hg^2+^ is often detected in water systems, pH 7.0 and H_2_O/CH_3_OH 97:3, v/v was selected as the solvent system for the detection.

### Study on the mechanism of the OST as a fluorescent Hg^2+^ probe

#### The binding ratio of the OST–Hg^2+^ complex

**Figure 5:**
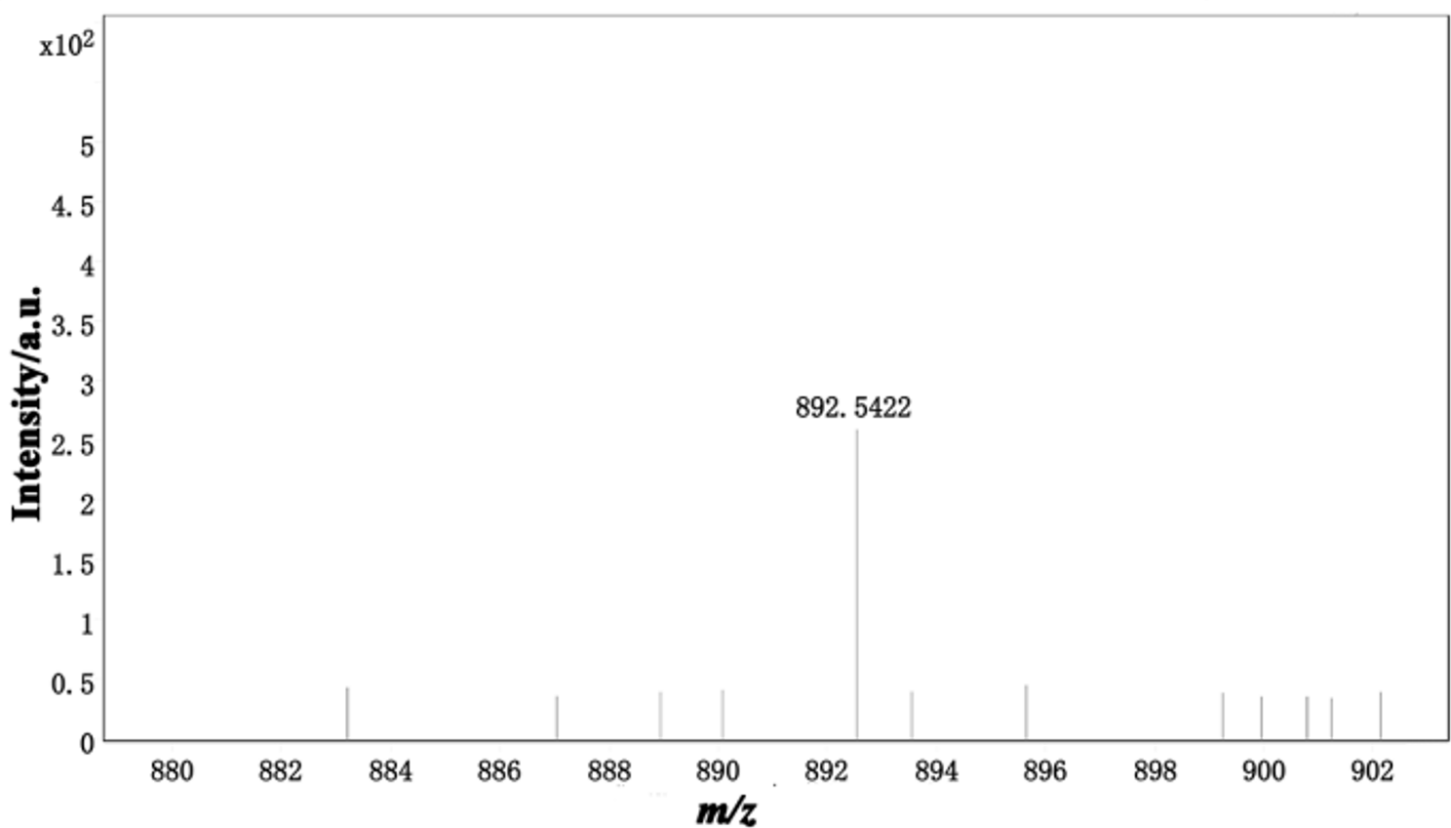
Mass spectrum of the probe with Hg^2+^.

The stoichiometric ratio between OST and Hg^2+^ was determined using the Job method (constant mole variation), and the results are shown in Figure S3, Supporting Information File 1. The maximum UV absorption intensity was achieved at *n*(Hg^2+^)/*n*((OST) + (Hg^2+^)) = 0.5 at 325 nm, indicating that the binding ratio of the OST probe to Hg^2+^ was 1:1. Combined with mass spectrometry data (Figure 5), the fragment peak of ESIMS at *m*/*z* 892.5422 corresponded to [OST_2_–Hg_2_]^+^ (calculated as 892.5429), indicating that OST and Hg^2+^ formed a 2:2 complex.

#### ^1^H NMR titration analysis of the mode of interaction between the probe and Hg^2+^

**Figure 6:**
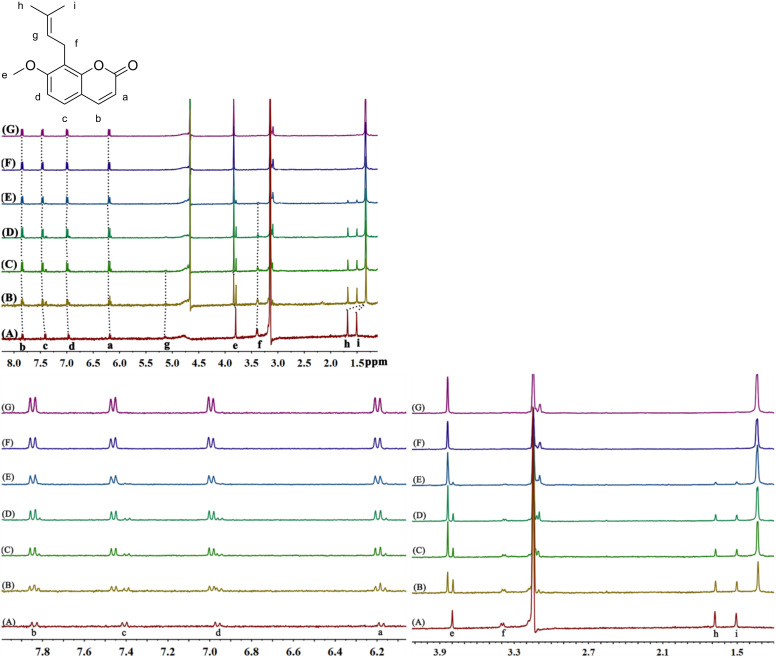
^1^H NMR titration spectra recorded for OST (*c* = 5 × 10^−4^ mol∙L^−1^) during the addition of different molar equivalents of Hg^2+^ (400 MHz, D_2_O, *n*(Hg^2+^)/*n*(OST) = 0 (A), 0.2 (B), 0.4 (C), 0.8 (D, E), 1.0 (F), 1.2 (G), pH 7.0, D_2_O/CH_3_OH 97:3, v/v).

The chemical shift of the resonance peak between the OST probe and Hg^2+^ in a ^1^H NMR titration experiment was used to infer the binding mode of the probe and Hg^2+^. Figure 6 shows a ^1^H NMR titration of OST in the presence of Hg^2+^ at different concentrations. With the addition of Hg^2+^, the OST proton resonances shift. At *n*(Hg^2+^)/*n*(OST) = 1, the chemical shift of the proton signals did not change, indicating that the interaction ratio of OST and Hg^2+^ was 1:1. At this time, the signals of the protons H_a_, H_b_, H_c_, H_d_, and H_e_ shifted downfield by 0.02, 0.03, 0.06, 0.04, and 0.04 ppm, respectively. This was due to the formation of the OST–Hg^2+^ complex, and the oxygen atoms on the aromatic ring were complexed by Hg^2+^, and thus enhancing the electron absorption effect on the aromatic ring, reducing the electron cloud density, leading to the proton signal of the aromatic ring moving to a lower field. The signals of the protons H_f_, H_h_, and H_i_ were shifted to a higher field by 0.02, 0.35, and 0.18 ppm, respectively. The formation of the OST–Hg^2+^ complex decreased the distance between the two aromatic rings so that the protons H_f_, H_h_, and H_i_ on the branched chain were in the shielding region of the benzene ring and their signals shifted to a higher field. When the protons H_h_ and H_i_ of the two methyl groups were in the shielding region of the aromatic ring, the chemical shifts moved to a higher field. Because H_h_ is located in the central area of the shield and H_i_ is at the edge of the shield, the Δδ value of H_h_ is relatively large, and finally both moved to δ 1.35.

**Figure 7:**
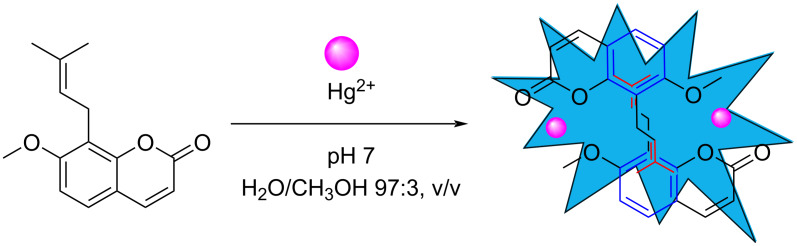
The binding mode of OST and Hg^2+^.

Based on the ^1^H NMR and mass spectrometry data, combined with the analysis of the UV–vis spectrum, the interaction mode between OST and Hg^2+^ is suggested as shown in Figure 7. Hg^2+^ and the oxygen atoms of the lactone ring and the methoxy group formed a stable complex, resulting in the enhanced fluorescence.

#### Isothermal titration calorimetry analysis

A Hg^2+^ solution (2 × 10^−3^ mol∙L^−1^, 25 μL) was added to the 1 × 10^−4^ mol∙L^−1^ OST probe solution at 25 °C to record the exothermic binding isotherms [28], and the measurement results obtained are shown in Figure S4 and Table S1, Supporting Information File 1. By the formula Δ*G* = −*R* ⋅ *T* ⋅ ln(*K*_a_) = Δ*H − T* ⋅ Δ*S*, with Δ*H* < 0 and *T* ⋅ Δ*S* < 0, *ΔG* was determined to be <0, and therefore the thermodynamic parameters show that the binding process between OST and Hg^2+^ was mainly determined by Δ*H*, with an action ratio of 1:1 and a *K* value of 1.552 × 10^5^ L ∙ mol^−1^.

### Measurement of the fluorescence quantum yield

The fluorescence quantum yield of ʟ-tryptophan at an excitation wavelength of 293 nm was 0.14, which was taken as the standard (Figure S5, Supporting Information File 1) [29–30]. The integration range of ʟ-tryptophan was 280–540 nm and that of OST–Hg^2+^ was 300–540 nm, and the fluorescence quantum yield of the aqueous probe solution was measured to be 0.08. Although this quantum yield is not high, the selectivity and sensitivity for the fluorescence analysis are good. This can be better used to detect Hg^2+^ ions and has a certain analytical value.

### Determination of Hg^2+^ in a water sample

Tap water was directly used for the determination of Hg^2+^ using the standard addition method, and the recovery rate of the sample was determined. The results are listed in Table 1.

**Table 1:** Determination of Hg^2+^ in tap water.^a^

Hg^2+^ (μmol∙L^−1^)	detected (mean ± SD, μmol∙L^−1^)	recovery rate (%)	RSD (*n* = 3, %)

0	–	–	–
12	11.95	99.58	2.63
16	15.86	99.13	0.31
18	17.67	98.17	0.65

^a^SD = standard deviation, RSD = relative standard deviation.

As shown in Table 1, the probe can not only identify Hg^2+^ but also effectively detect Hg^2+^ in tap water, which further proves that the applicational value of the probe is considerable.

## Conclusion

In this paper, OST was used as the fluorescent probe to establish a new method for the determination of Hg^2+^. Within the range of 6.0 × 10^−5^ to 24.0 × 10^−5^ mol∙L^−1^, the change in the fluorescence intensity of the system had a good linear relationship with the concentration of mercury ions. The linear equation was *y* = 664.91 ∙ *x* − 94.92, the detection limit was 5.04 × 10^−8^ mol∙L^−1^, and the recognition process was the result of a chelation fluorescence enhancement mechanism. The method is simple to be executed, has a high sensitivity and good selectivity, and can be used to quantitatively detect and monitor mercury ions in the environment.

## Supporting Information

File 1 General information and descriptions of the methods.

## References

[1] Ullrich S M, Tanton T W, Abdrashitova S A (2001). Crit Rev Environ Sci Technol.

[2] Regnell O, Watras C J (2019). Environ Sci Technol.

[3] Li Y, Zhao J, Zhong H, Wang Y, Li H, Li Y-F, Liem-Nguyen V, Jiang T, Zhang Z, Gao Y (2019). Environ Sci Technol.

[4] Fong B M W, Siu T S, Lee J S K, Tam S (2007). J Anal Toxicol.

[5] Yuan H, Gao S, Liu X, Li H, Günther D, Wu F (2004). Geostand Geoanal Res.

[6] Rastogi L, Dash K, Arunachalam J (2013). J Pharm Anal.

[7] Macka M, Haddad P R (1997). Electrophoresis.

[8] Du J, Hu M, Fan J, Peng X (2012). Chem Soc Rev.

[9] Formica M, Fusi V, Giorgi L, Micheloni M (2012). Coord Chem Rev.

[10] Chen X, Pradhan T, Wang F, Kim J S, Yoon J (2012). Chem Rev.

[11] Quang D T, Kim J S (2010). Chem Rev.

[12] Wang X, Ma X, Wen J, Geng Z, Wang Z (2020). Talanta.

[13] YouMing Z, BingBing S, Peng Z (2013). Sci China: Chem.

[14] Xu Y, Jiang Z, Xiao Y, Zhang T-T, Miao J-Y, Zhao B-X (2014). Anal Chim Acta.

[15] Hennrich G, Walther W, Resch-Genger U, Sonnenschein H (2001). Inorg Chem.

[16] Yang M-H, Thirupathi P, Lee K-H (2011). Org Lett.

[17] Jing S, Zheng C, Pu S, Fan C, Liu G (2014). Dyes Pigm.

[18] Pan Z, Fang Z, Lu W, Liu X, Zhang Y (2015). J Ethnopharmacol.

[19] Lian Q (2003). Zhongyaocai.

[20] Luszczki J J, Wojda E, Andres-Mach M, Cisowski W, Glensk M, Glowniak K, Czuczwar S J (2009). Epilepsy Res.

[21] Liang H-J, Suk F-M, Wang C-K, Hung L-F, Liu D-Z, Chen N-Q, Chen Y-C, Chang C-C, Liang Y-C (2009). Chem-Biol Interact.

[22] Zhang H, Zhang H, Qu C, Bai L, Ding L (2007). Spectrochim Acta, Part A.

[23] Zhao H, Song F, Zhang J, Wang F, Liu J, Liu Y (2013). J Opt Soc Am B.

[24] Lin Q, Fu Y-P, Chen P, Wei T-B, Zhang Y-M (2013). Dyes Pigm.

[25] Wang H-H, Xue L, Yu C-L, Qian Y-Y, Jiang H (2011). Dyes Pigm.

[26] Wen J, Geng Z, Yin Y, Zhang Z, Wang Z (2011). Dalton Trans.

[27] Ding S-Y, Dong M, Wang Y-W, Chen Y-T, Wang H-Z, Su C-Y, Wang W (2016). J Am Chem Soc.

[28] O’Neill M A A, Gaisford S (2011). Int J Pharm.

[29] de Lucas N C, Santos G L C, Gaspar C S, Garden S J, Netto-Ferreira J C (2014). J Photochem Photobiol, A.

[30] Wohlgemuth M, Bonačić-Koutecký V, Mitrić R (2011). J Chem Phys.

