# Supplementary material for: The fluorescence of a mercury probe based on osthol
Source: Beilstein J Org Chem. 2021 Jan 5;17:22–7. doi: 10.3762/bjoc.17.3 (PMC7801779; doi:10.3762/bjoc.17.3)
Supplement: File 1 — General information and descriptions of the methods. [file Beilstein_J_Org_Chem-17-22-s001.pdf]

BEILSTEIN JOURNAL OF ORGANIC CHEMISTRY

Supporting Information

for

The fluorescence of a mercury probe based on osthol

Guangyan Luo, Zhishu Zeng, Lin Zhang, Zhu Tao and Qianjun Zhang

Beilstein J. Org. Chem. **2021,** *17,* 22–27. doi:10.3762/bjoc.17.3

General information and descriptions of the methods

License and Terms: This is a supporting information file under the terms of the Creative Commons Attribution License (https://creativecommons.org/licenses/by/4.0). Please note that the reuse, redistribution and reproduction in particular requires that the author(s) and source are credited and that individual graphics may be subject to special legal provisions.
The license is subject to the *Beilstein Journal of Organic Chemistry* terms and conditions: (https://www.beilstein-journals.org/bjoc/terms)

Table of contents

Experimental S1

Reagents S1

Instruments S2

Methods S2

OST fluorescence probe selectivity to metal ions S2

Interference and competitive experiment of metal ions S3

OST probe fluorescence titration of mercury S3

^{1}H NMR and MS spectroscopy S3

UV–vis measurements S5

Isothermal titration calorimetry (ITC) measurements S5

Measurement of the fluorescence quantum yield S6

References S7

Experimental

Reagents

Osthol (analytical purity ≥ 98%) was purchased from Shanghai Yuanye Biochemical Technology Co., Ltd. The water is the secondary resteaming water. Methanol was analytical pure. Hydrochloric acid (analytical purity ≥ 99.5%) was purchased from Tianjin Kaixin Chemical Industry Co., Ltd. Deuterated heavy water (analytical purity ≥ 99.9%) was purchased from SAAN Chemical Technology (Shanghai) Co., Ltd.

Mercury nitrate was purchased from Aladdin Ltd. Other metal perchlorates of

Na^+, K^+, Al^{3+}, Ca^{2+}, Fe^{2+}, Ba^{2+}, Cu^{2+}, Ag^+, Fe^{3+}, Cr^{3+}, Mn^{2+}, Cd^{2+}, Ni^{2+}, Zn^{2+}, Mg^{2+}, CO^{2+}, and Pb^{2+} were purchased from Aladdin Co., Ltd.

Instruments

A UV-2700 dual-beam UV–vis spectrophotometer (Shimadzu Instruments Co., Ltd.), VARIANCARYE-CLIPSE fluorescence spectrophotometer (Varian, USA), Agilent 6545 Q-TOF LC/MS, PHS-25 digital pH meter (Shanghai INESA Scientific Instrument Co., Ltd.), AKHL-III-08 Eco laboratory ultrapure water machine (Chengdu Eco Water Treatment Equipment Co., Ltd.), FA2204N electronic balance (Shanghai Jinghai Instrument Co., LTD.), Nano ITC isothermal calorimeter (TA company, USA), JNM-ECZ400s MHz NMR system (JEOL), SHA-IIIS constant temperature oscillator (Zhengzhou Great Wall Technology Industry and Trade Co., Ltd.), and 101-1AB electric heating blast drying oven (Tianjin Test Instrument Co., Ltd.) were used.

Methods

OST fluorescence probe selectivity to metal ions

A VARIANCARYE-CLIPSE fluorescence spectrophotometer was used to measure the selectivity to the metal ions. Secondary water was used as the solvent. All the metal ions were prepared as a 0.2 mol · L^{-1} stock solution. Methanol was used as the solvent to prepare the OST probe reserve solution with a concentration of 1.0×10^{-3} mol·L^{-1}. At pH 7.0, a H_2O/CH_3OH 97:3, v/v solution of the OST (3.0×10^{-5} mol·L^{-1}) was prepared for characterization. To obtain fluorescence spectra, known quantities of ion solutions were added to

the OST. In aqueous solution, the fluorescence spectra were obtained by excitation at 325 nm, with 10 nm emission and excitation bandwidths, and the emission intensity was monitored at 300–540 nm at room temperature [1].

Interference and competitive experiment of metal ions

3 ml of a 3.0×10^{-5} mol·L^{-1} H_2O/CH_3OH 97:3, v/v solution of the OST probe at pH 7 and an equimolar concentration of Hg^{2+} were added into a quartz fluorescence cuvette. Then, added 1.2×10^{-4} mol·L^{-1} of various metal ions to the OST–Hg^{2+} system. The excitation wavelength was 325 nm and the slit was 10:10. Fluorescence measurement was carried out under the conditions [2].

OST probe fluorescence titration of mercury

3 mL of a 3.0×10^{-5} mol·L^{-1} solution of the OST probe were added into a quartz fluorescence cuvette with a width of 1 cm, and then $n(Hg^{2+})/n(OST)$ = 0, 0.2, 0.4, 0.6, 0.8, 1.0, 1.2, 1.4, 1.6, 1.8, 2.0, respectively, were added, mixed well, and the fluorescence intensity was detected. The excitation wavelength was 325 nm and the slit was 10:10. Fluorescence measurements were carried out under the conditions found in Reference [3].

^{1}H NMR and MS spectroscopy

A JEOL JNM-ECZ400s spectrometer was used to record the ^{1}H NMR spectra at 25 °C. ^{1}H NMR titration experiments of OST with Hg^{2+} were performed in D_2O/CD_3OD 97:3, v/v. That is, the concentration of OST was set as 5.0×10^{-4} mol·L^{-1}, and Hg^{2+} was gradually added dropwise to the solution by used of the molar ratio method.

ESITOF mass spectra of the OST–Hg^{2+} complex were recorded on an Agilent 6545 Q-TOF spectrometer at room temperature. For that, a mixed alcohol/water solution of OST–Hg^{2+} was prepared at a concentration of 1.0×10^{-4} mol·L^{-1}. Then, the solution was filtered and tested by MS.

Impact of the pH value of the solution

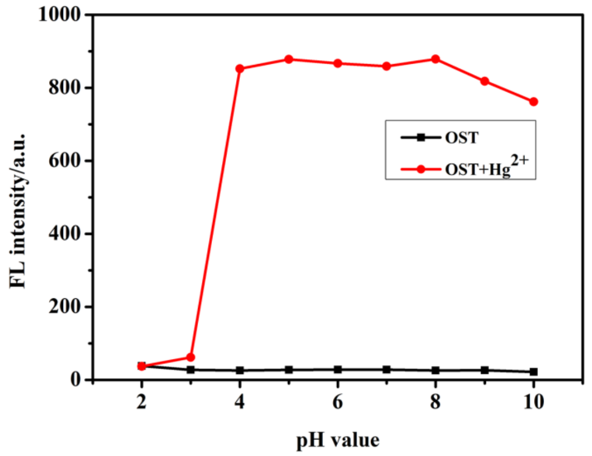

Figure S1: Effects of different pH values on OST and OST–Hg^{2+}.

Effect of the solvent

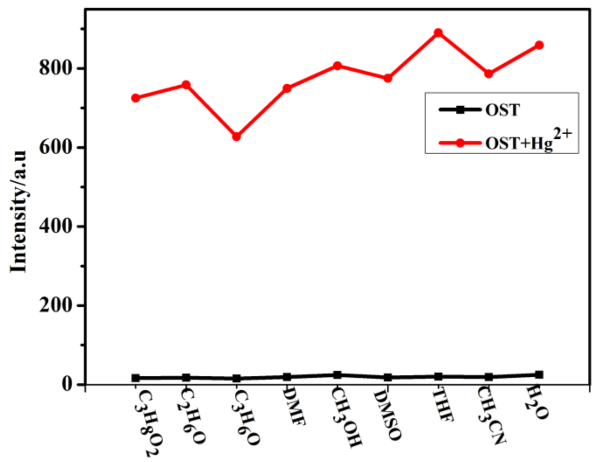

Figure S2: Fluorescence spectra of OST–Hg^{2+} (3.0×10^{-5} mol·L^{-1}) in different solvents.

UV–vis measurements

UV–vis spectra of samples in 1 cm quartz cells were recorded on a UV-2700 double beam UV–vis spectrophotometer. The equimolar continuous change method (Job method) was used to determine the binding ratio. A series of solutions of *n*(OST)/*n*(OST + Hg^{2+}) = 0, 0.1, 0.2, 0.3, 0.4, 0.5, 0.6, 0.7, 0.8, 0.9, and 1.0, respectively, was prepared, and the corresponding solutions were used without Hg^{2+} as blank controls. The absorption was measured at room temperature.

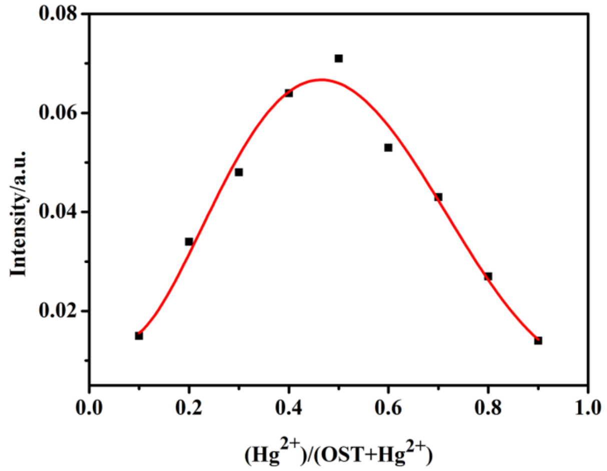

Figure S3: The Job plot data of the probe OST with Hg^{2+} present.

Isothermal titration calorimetry (ITC) measurements [4]

Thermodynamic parameters were determined by the isothermal titration calorimeter Nano ITC (TA, USA). An aqueous solution of Hg^{2+} with a concentration of 2.0×10^{-3} mol·L^{-1} and a solution of OST (pH = 7, containing 1.0% methanol) with a concentration of 1.0×10^{-3} mol·L^{-1} were prepared and mixed well before used. The OST probe solution was titrated with the Hg^{2+}

solution to determine the binding constant. The measurement conditions used on the Nano ITC device were: 25 °C, 6 μL·d^{-1}, the interval time was 250 s, and the stirring speed was 250 r·min^{-1}.

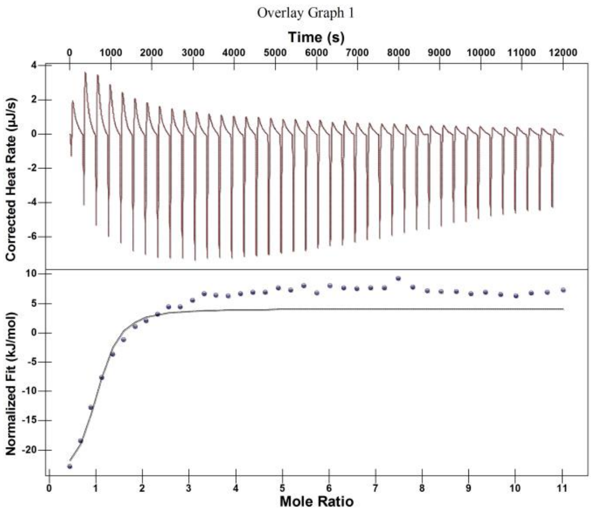

Figure S4: The interaction between the OST probe and Hg^{2+}.

Table S1: Thermodynamic binding data for OST–Hg^{2+}.

Hg^{2+}	*K* (L · mol^{-1})	Δ*H* (kJ · mol^{-1})	*n*	*T* · Δ*S* (kJ · mol^{-1})	K_D (L ·mol^{-1})
OST	1.552×10^5	−29.70	0.972	0.065	6.441×10^{-6}

Measurement of the fluorescence quantum yield

The fluorescence quantum yield (Y) is the ratio of the number of photons emitted by the fluorescent material after absorbing light to the number of photons of the excited light absorbed. The fluorescence quantum yield was measured by the reference method. By measuring the integral fluorescence intensity and the absorbance of the diluted solution of the tested substance and the reference substance at the same excitation wavelength, the fluorescence

quantum yield of the tested substance was calculated according to a formula from Reference [5]: Yu = Ys · Fu/Fs · As/Au. Among them, Yu and Ys represent the fluorescence quantum yield of the test substance and the reference standard substance, respectively. Fu and Fs represent the comprehensive fluorescence intensity of the test product and the reference product, respectively. Au and As represent the absorbance of the incident light at the excitation wavelength of the test substance and the reference substance, respectively (A = ε · *b* · *c*).

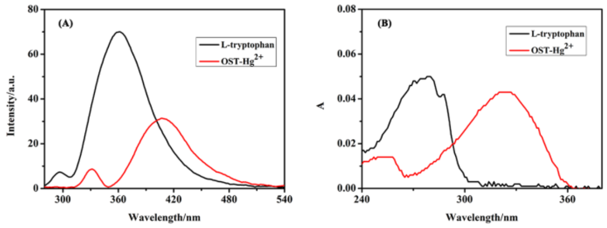

Figure S5: Fluorescence spectra of (A) ʟ-tryptophan and OST–Hg^{2+} and (B) UV−vis absorption spectra.

References

1. Wang, X.; Ma, X.; Wen, J. *Talanta*, **2019,** *207,* 120-311.

2. Lin, Q.; Fu, Y.P.; Chen, P. *Dyes & Pigments*, **2013,** *96,* 1-6.

3. Wen, J.; Geng, Z.; Yin, Y. *Dalton Trans*, **2011,** *40,* 1984-1989.

4. Michael A, A.; O'Neill, Gaisford, S. *Int J Pharm,* **2011,** *417,* 83-93.

5. Kumari, A ,; Kumar, A.; Sahu, S. K. *Sensors & Actuators B Chemical*, **2017,** *254,* 197-205.
